# Sudden unexpected death in epilepsy: Investigation of autopsy-based studies

**DOI:** 10.3389/fneur.2023.1126652

**Published:** 2023-02-16

**Authors:** Fengping Yan, Fu Zhang, Yanan Yan, Le Zhang, Yuanyuan Chen

**Affiliations:** ^1^Department of Forensic Medicine, School of Basic Medical Sciences, Gannan Medical University, Ganzhou, Jiangxi, China; ^2^Key Laboratory of Prevention and Treatment of Cardiovascular and Cerebrovascular Disease of Ministry of Education, Gannan Medical University, Ganzhou, Jiangxi, China; ^3^Key Laboratory of Forensic Pathology, Ministry of Public Security and Criminal Technology Center of Guangdong Province Public Security Bureau, Guangzhou, Guangdong, China; ^4^Forensic Center of Gannan Medical University, Ganzhou, Jiangxi, China

**Keywords:** epilepsy, SUDEP, autopsy, cause of death, forensic pathology

## Abstract

Epilepsy is a common neurological disorder that is associated with increased morbidity and mortality. Sudden unexpected death in epilepsy (SUDEP) is one of the most common causes for epilepsy-related deaths and its characteristics remain largely unknown, particularly from a forensic autopsy perspective. The present study aimed to investigate the neurological, cardiac, and pulmonary findings for a total of 388 SUDEP decedents, encompassing three cases from our forensic center during 2011–2020 and 385 literature-reported autopsy cases. In the cases mentioned in this study, two of them presented with only mild cardiac abnormalities, such as focal myocarditis and mild coronary atherosclerosis of the left anterior coronary artery. The third one was negative of any pathological findings. After pooling together these SUDEP cases, we found that neurological changes (*n* = 218 cases, 56.2%) were the most common postmortem findings associated with SUDEP, with cerebral edema/congestion (*n* = 60 cases, 15.5%) and old traumatic brain injury (*n* = 58 cases, 14.9%) being the major findings. Interstitial fibrosis, myocyte disarray/hypertrophy, and mild coronary artery atherosclerosis were the most common findings related to primary cardiac pathology, documented in 49 (12.6%), 18 (4.6%), and 15 (3.9%) cases, respectively. Non-specific pulmonary edema was the major finding in the lungs. This is an autopsy-based study that reports the scenario of postmortem findings for SUDEP cases. Our study paves the way for understanding the pathogenesis of SUDEP and the interpretation of death.

## 1. Introduction

Sudden unexpected death in epilepsy (SUDEP) was defined as “sudden, unexpected, witnessed or un-witnessed, non-traumatic and non-drowning death in patients with epilepsy, with or without evidence for a seizure and excluding documented status epilepticus, in which postmortem examination does not reveal a toxicological or anatomic cause for death” (1). It is considered the main cause of death in patients with epilepsy and is the second most common neurological cause for potential years of life lost among all neurological diseases, second only to stroke (2, 3). The incidence rate of SUDEP was reported as 1.16 cases per 1,000 patients with epilepsy. SUDEP affected all age groups, but primarily young people with its incidence in the 20–45-year age group being 27 times higher than in control groups (4). Several risk factors have been identified, with generalized tonic–clonic seizures as the most important one. Other key risk factors include high seizure burden, lack of antiepileptic drug treatment, poly-therapy, intellectual disability, and prone position at the time of death (5).

In suspected SUDEP cases, a complete postmortem examination including both external and full internal examination, as well as toxicological analysis of antiepileptic drug (AED) levels should be made mandatory. Unfortunately, at postmortem investigation, a proportion of SUDEP cases were often unwitnessed and there was no information available on the victim's last moment of life and on the possible clues of seizures before death. Moreover, the majority of SUDEP cases were absent or at subtherapeutic levels of all AEDs (6). Owing to the limited evidence, it is often difficult to clarify whether epilepsy was the cause of death or not. Published case series did not suggest any definite pathological features or biomarkers for diagnosis of SUDEP (7). The most common mechanisms being studied are neuro-cardio-respiratory connections since ictal activity that arises in or spreads to the central autonomic network can disrupt functional connectivity of its network by inhibiting or activating autonomic areas, causing diverse autonomic manifestations, including cardiovascular and respiratory dysfunction, and brainstem damage (8, 9). There is also considerable evidence indicating that genetic factors may play a role. Cardiac genes associated with long QT syndrome, bradycardia, and sudden cardiac death can cause both epilepsy and arrhythmias or increase the risk of seizure-induced arrhythmias and have been linked to SUDEP (10, 11). Moreover, some AEDs may worsen patients' conditions, leading to other health complications including cardiovascular dysfunctions such as myocardial infarction, arrhythmias, and even cardiovascular death or SUDEP (12). All these studies provide a potential understanding of the mechanisms behind the SUDEP. However, the etiology and definite pathogenic mechanisms leading to SUDEP are still unknown.

The limited understanding of SUDEP pathogenesis is, at least in part, due to the scarce knowledge of postmortem findings for these cases. We present three cases of SUDEP individuals and summarized their epidemical and forensic characteristics. We also searched the literature on SUDEP that provided postmortem examination data, thereby summarizing common neurological, cardiac, or pulmonary pathologies with all these SUDEP cases, for the purpose of providing further insight into SUDEP from a forensic autopsy perspective.

## 2. Materials and methods

### 2.1. Study design

This is a retrospective and descriptive study with research interest in SUDEP cases undergoing a full autopsy examination. The study method was similar to those mentioned in previous studies (13, 14). We initially collected authentic SUDEP cases from our single center and then searched published literature to gather all relevant cases. We then described the neurological, cardiac, and pulmonary findings based on all the available SUDEP cases.

### 2.2. Case collection

The cases were collected between January 2011 and December 2020 in the Forensic Center of the Gannan Medical University. Data for this study were taken from the completed postmortem reports, including details of sex, age at death, scene at death, circumstances surrounding the event, autopsy findings, and postmortem toxicological results. Cases were collected based on the following criteria: (1) decedents had a clear record of epilepsy or seizure disorder; (2) decedents experienced a sudden, unexpected, witnessed or unwitnessed, non-traumatic, and non-drowning death; and (3) absence of definite anatomic or toxicological cause of death after complete examination.

Death certificate for these patients was made without controversy by three independent pathologists. In case of multiple pathological changes, the severity of each pathological change and its contribution to the death were seriously evaluated and independently decided by three pathologists. In case of suspected SUDEP, a neuropathologist was routinely consulted. In case of inconsistent conclusion, the case was consulted with another external pathologist to reach the final decision.

Each case was anonymized to protect the patient's privacy. This study only extracted patients' information from archived records without using patients' specimens. The review of patients' medical and forensic records was approved by the Ethical Review Board at the School of Basic Medical Science, Gannan Medical University (Approval No.: 2022-178).

### 2.3. Literature search strategy and selection criteria

To collect the most matched literature, we used a two-step screen strategy, to systematically obtain publications reporting on cases of patients with epilepsy who died suddenly and unexpectedly and underwent autopsy. Initially, we used terms such as “Epilepsy” and “autopsy” or “SUDEP” and “autopsy” to detect all publications that studied the SUDEP cases from a forensic autopsy perspective. The search was limited to articles published in the English language. The restriction in publication date was set from January 1980 to September 2022.

After initial screening, candidate articles were further evaluated by title and abstract, and then by full-text reading. Studies that did not report postmortem findings were excluded. Reference lists of the retrieved studies were also checked for potential additional articles. Types of studies include retrospective study, case-control study, prospective study, survey, and case report, only if they provided macroscopic and microscopic results of the brain, the heart, and the lungs, as well as toxicological results including serum AED concentrations.

## 3. Result

### 3.1. Basic characteristics and autopsy findings of the three SUDEP cases

A review of the files from our forensic center yielded eight cases that had a clear medical history of epilepsy. Among these, three (three out of eight) cases were identified to have died from SUDEP, the other five cases died from explainable causes, such as accidental drowning (*n* = 1), suicide (*n* = 1), status epilepticus (*n* = 2), and rupture of the aortic dissection (*n* = 1). Of the three SUDEP cases aged 30.5 ± 7.3 years, two were men and one was a woman. The etiology of epilepsy is unclear for all three cases. Two individuals died at their residence, unwitnessed, found in bed or on the bedroom floor in a prone position. The third individual was witnessed to die during the daytime when watching TV. At autopsy, no significant toxicological or anatomical findings were revealed, except bite marks on the tongue and lips for two cases. Periorbital bruise was found in the third individual, indicating potential injury possibly caused by epileptic seizure prior to death. Two cases were presented with mild cardiac abnormalities, such as focal myocarditis and mild coronary atherosclerosis of the left anterior coronary artery. These two subjects also had detectable AEDs at the time of death, both in a sub-therapeutic range.

Basic characteristics and autopsy findings of the three cases categorized as SUDEP are summarized in Table 1.

**Table 1:** Basic characteristics and autopsy findings of the three sudden unexpected deaths in epilepsy (SUDEP) cases.

	**Age**	**Sex**	**Etiology detail for epilepsy**	**Medical history**	**Circumstances of death**	**Autopsy findings at postmortem**	**Postmortem toxicology**
1	19	Male	Childhood-onset epilepsy (since 6–8 years old)	He had collapsed and lost consciousness 2 weeks before death. On a regular carbamazepine treatment	Unwitnessed, found dead in the morning in his bed, in a prone position	**Macroscopy:** tongue bite mark	Sub-therapeutic (carbamazepine)
						**Microscopy:** pulmonary congestion, minimal focal myocarditis, no remarkable neuropathological abnormality	
2	36	Male	Unknown	He was a current smoker and had chronic seizure disorder with EEG positivity. Lists of AEDs medication unknown	Unwitnessed, found dead in the morning on his bedroom floor, in a prone position	**Macroscopy:** lip bite mark.	Sub-therapeutic (valproic acid)
						**Microscopy:** pulmonary congestion, atherosclerosis of the left anterior descending coronary artery (Grade 1) without acute or old myocardial ischemia, without remarkable neuropathological abnormality	
3	34	Female	Unknown	She had a history of hypertension and chronic seizure disorder with EEG positivity. List of cardiovascular and AEDs medication unknown	Witnessed, had a witnessed seizure when watching TV during the daytime, Cardiopulmonary Resuscitation failed	**Macroscopy:** periorbital bruise, and resuscitation marks	Negative
						**Microscopy:** cerebral edema, and mild myocardial fibrosis, without typical pathological changes of hypertensive heart disease	

### 3.2. Results of the literature search

The described search strategy yielded 16 publications, including 7 retrospective studies, 3 case-control studies, 5 case reports, and 1 prospective study, claiming 385 SUDEP cases in total (15–30). These studies were mostly from the USA (9/16). Among the 385 SUDEP cases, the male-to-female ratio was 227:158, similar to the cases reported in this study. Cases from the published literature are concentrated in the age group of 10–40 years, with the age range from 8 months to 83 years. Information on these studies and their detailed findings are documented in Table 2.

**Table 2:** Summary of the postmortem findings from the autopsy study series.

	**References (region)**	**Type of study**	**Patients** ** Number of cases (*N*), gender, age range**	**Autopsy findings (neurological, cardiac, and pulmonary pathologies)**	**Postmortem AED levels**
1	Terrence et al. (15), USA	Retrospective study	SUDEP cases underwent postmortem examination during the calendar years 1978 and 1979 recorded by the Allegheny Country Coroner's Office, Pittsburgh, PA *N* = 8 (4 males, 4 females, age range: 9–31 years)	**Neuropathology:** 2 with cerebral edema, 1 with old trauma to the brain. **Cardiac pathology:** no detail. **Pulmonary pathology:** all with pulmonary congestion/ edema	Sub-therapeutic: 2; Therapeutic: 3; Negative: 3
2	Leestma et al. (16), USA	Prospective study	SUDEP cases undergoing examination of the brain by the Medical Examiner of Cook Country (Chicago, IL) in the year 1983 *N* = 60 (46 males, 14 females, age range: 8 months−83 years)	**Neuropathology:** 24 with old contusions or traumatic brain injury (including old contusion, old penetrating injury, chronic subdural hematoma, and meningeal fibrosis), 7 with Ammon's horn sclerosis, 6 with hydrocephalus, 4 with cortical malformation, 4 with diffuse cerebellar degeneration, 1 with arteriovenous malformation, 1 with cerebral hemiatrophy, 1 with brain tumor, 8 with old cerebrovauclar accident; 14 with cerebral edema. **Cardiac pathology:** 9 with mild to moderate atherosclerosis; 2 with myocardial fibrosis. **Pulmonary pathology:** 42 with pulmonary congestion/edema	Sub-therapeutic: 51; Therapeutic: 3
3	Earnest et al. (17), USA	Retrospective study	“Sudden Unexplained Death Syndrome” cases identified in autopsy reports of persons with epilepsy from the Coroner's office of Denver Country and four adjacent counties from Jan 1982 through June 1987 *N* = 44 (28 males, 16 females, age range: 3–58 years)	**Neuropathology:** 14 with cerebral edema. **Cardiac pathology:** 5 with micro-focal interstitial fibrosis. **Pulmonary pathology:** 38 with pulmonary congestion/edema	Sub-therapeutic: 35; Therapeutic: 3; No detail: 6
4	Natelson et al. (18), USA	Case-control study	SUDEP cases underwent autopsy with a careful pathologic evaluation of the hearts *N* = 7 (5 males 2 females, age range: 12–44 years)	**Neuropathology:** 1 with communicating hydrocephalus. **Cardiac pathology:** 5 with myocyte vacuolization, 4 with interstitial fibrosis. **Pulmonary pathology:** no detail	Therapeutic: 2; No detail: 5
5	Antoniuk et al. (19), Brasil	Retrospective study	Cases recognized as SUDEP among deaths registered between Jan 1990 to July 1999 that underwent postmortem examination at the medico-legal institute of Crutibit-Brazia *N* = 20 (14 males, 6 females, age range: 17–47 years)	**Neuropathology:** 7 with cerebral edema. **Cardiac pathology:** no detail; **Pulmonary pathology:** 8 with pulmonary edema, 1 with pulmonary hemorrhage	No details
6	Shields et al. (20), USA	Case-control study	SUDEP cases between 1996–2000 underwent gross examination of the brain by either a forensic pathologist or consulting neuro-pathologist. Focusing on neuro-pathological findings in SUDEP cases *N* = 70 (38 males, 32 females, age range: 16–71 years)	**Neuropathology:** 19 with traumatic event (including contusions, gliosis, necrosis, cystic encephalomalacia, gunshot wound, and previous craniotomy site), 9 with cortical atrophy, 10 with cerebellar atrophy, 2 with venous hemangioma, 1 with leptomeningeal varix, 1 with tumor. **Cardiac pathology:** no details. **Pulmonary pathology:** 56 with pulmonary edema/congestion	Sub-therapeutic:29; Therapeutic: 13; Supra-therapeutic: 1; Negative: 27
7	Swallow et al. (21), UK	Case report	A case of 18-year-old boy died of SUDEP with postmortem examination from The Welsh Epilepsy Unit, University Hospital of Wales	Autopsy showed extensive cerebral edema and infarction, lungs were heavily congested and oozed edema, no details of cardiac pathology	No detail
8	Simona et al. (22), Denmark	Case-control study	Autopsy cases of SUDEP individuals during the period Jan 1998 to Sep 2000 by the department of Forensic Medicine, Aarhus University *N* = 15 (6 males, 9 females, age range: 14–58 years)	**Neuropathology:** no detail. **Cardiac pathology:** 6 with multiple foci of varying degrees of fibrosis in the myocardium. **Pulmonary pathology:** no detail	Sub-therapeutic: 7; therapeutic: 2; supra-therapeutic: 1; negative: 5
9	Pollanen et al. (23), Canada	Retrospective study	SUDEP cases identified from March 2005 through May 2010 in the Provincial Forensic Pathology Unit, Toronto, Ontario, Canada *N* = 24 (11 males, 13 females, age range: 19–65 years)	**Neuropathology:** 1 with hippocampal sclerosis, 4 with old cortical contusions, 1 with gliotic scar, 1 with neuronal migration disorder. **Cardiac pathology:** no detail. **Pulmonary pathology:** no detail	Sub-therapeutic OR negative: 15; therapeutic: 8; supra-therapeutic: 1; unknown: 1
10	Zhuo et al. (24), USA	Retrospective study	Forensic autopsy SUDEP cases from 2007 to 2009 at the Office of the Chief Medical Examiner in the State of Maryland *N* = 74 (43 males, 31 females, age range: 14–63 years)	**Neuropathology:** 10 with traumatic lesions, 5 with malformation of cortical development, 4 with cerebellar atrophy, 3 with vascular malformation, 4 with acute focal subarachnoid hemorrhage, 2 with hippocampal gliosis, 2 with cerebral and hippocampal atrophy, 2 with remote infarcts, 3 with brain tumor, 1 with capillary angioma, 1 with acute hypoxic-ischemia change of hippocampi, 1 with cerebellar sclerosis, 1 with tuberous sclerosis. **Cardiac pathology:** 7 showed moderate ventricular hypertrophy; 3 had mild atherosclerotic coronary artery disease, 22 showed varying degrees of focal fibrosis in the myocardium. **Pulmonary pathology:** 52 with pulmonary congestion/edema	Sub-therapeutic: 19; therapeutic: 6; supra-therapeutic: 1
11	Ryvllin et al. (25), USA	Retrospective study/survey	SUDEP cases with cardiorespiratory arrests between Jan 2008 and Dec 2009 from epilepsy monitoring units Located in Europe, Israel, Australia, and New Zealand *N* = 16 (7 males, 9 females, age range: 19–62 years)	**Neuropathology:** 2 with hippocampal atrophy, 2 with encephalitis, 1 with tumor. **Cardiac pathology:** 1 with sub-endocardial **f**ibrosis, 1 with coronary atherosclerosis. **Pulmonary pathology:** 3 with mild pulmonary edema	No details
12	Hashimoto et al. (26), Japan	Case report	A female student aged 19 died of SUDEP with postmortem examination from Department of Forensic Medicine, Graduate school of medicine, the University of Toyko	Brain edema, interstitial fibrosis, areteriolar wall thickening, and pulmonary edema with alveolar hemorrhage	No detail
13	Esen Melez et al. (27), Turkey	Retrospective study	Cases died of SUDEP in patients with a prior diagnosis of epilepsy, referred to the Ministry of Justice of Forensic Medicine in Istanbul between 2007 and 2011 *N* = 40 (21 males, 19 females, age range: 1–60 years)	**Neuropathology**: brain edema in 24. **Cardiac pathology**: hypertrophy in 11 cases, fibrosis in 9 cases. **Pulmonary pathology:** pulmonary edema in 37	Negative 21; Active 17 (without the details of concentration); Not performed 2
14	Neff and Lin (28), USA	Case report	A case of SUDEP of a 11-year-old girl with postmortem examination from Department of Laboratory Medicine and Pathology, Mayo Clinic	With focal myocardial infarct adjacent to bundle of His. No details of the neuro- and pulmonary pathology	Sub-therapeutic
15	Afandi et al. (29), Indonesia	Case report	A case of SUDEP of a 14-year-old boy with postmortem examination from Forensic Medicine and Medico-legal Studies Department, Faculty of Medicine, University Riau	With global cerebral edema and infarction, pulmonary edema, without remarkable change in the heart	Negative
16	Jordan et al. (30), USA	Case report	Three cases (a 33-year-old man, a 40-year-old man, and a 17-year-old girl) of SUDEP underwent postmortem examination from Department of Pathology, Wake Forest School of Medicine, Winstonsalem	**Neuropathology:** 1 with Schizencephaly, 1 with brain tumor, 1 with focal dysplasia. **Cardiac pathology:** 1 with coronary atherosclerosis, 2 with cardiomegaly. **Pulmonary pathology:** 1 with pulmonary edema	No details

### 3.3. Major neurological, cardiac, and pulmonary findings for the SUDEP cases

We then pooled our three cases with those publications, yielding a total of 388 cases (Table 3). After a full review of all these cases, we found that the cerebral edema/congestion and old traumatic injury were the most common symptoms of neurological pathology, accounting for 60 (15.5%) and 58 (14.4%) cases, respectively. Brain sclerosis, brain atrophy, cortical/vascular malformation, and old cerebrovascular infarction were the consequent changes, claiming 32 (8.3%), 14 (3.7%), 12 (3.1%), and 12 cases (3.1%), respectively. Intracranial tumors were found in 8 cases (2.1%). A total of 183 (47.2%) cases had none or unclarified neurological pathology.

**Table 3:** Major autopsy findings at the brain, the heart, and the lungs for sudden unexpected death in epilepsy (SUDEP) cases.

**Anatomic location**	**Pathology**	***N* (total = 388)**	**Percentage (%)**
Brain	Cerebral edema/congestion	60	15.5%
	Old traumatic brain injury/contusion	58	14.9%
	Encephal-atrophy (cortical/cerebral/cerebellar atrophy or cerebral degeneration)	32	8.3%
	Cortical/vascular malformations	14	3.7%
	Brain sclerosis (hippocampal sclerosis/cerebellar sclerosis/tuberous sclerosis/cerebral sclerosis)	12	3.1%
	Old cerebrovascular infarction	12	3.1%
	Intracranial tumors	8	2.1%
	Hydrocephalus	7	1.8%
	Encephalitis	3	0.8%
	Venous hemanioma	3	0.8%
	Others (acute hypoxic-ischamic changes 1, schizencehaly 1, focal dysplasia 1, glial scar 1, leptomeningeal varix 1, acute subarachnoid hemorrhage 4)	9	2.3%
	Non or unclarified	183	47.2%
Heart	Interstitial fibrosis	49	12.7%
	Myocyte disarray/hypertrophy	18	4.6%
	Mild atherosclerosis coronary artery disease	15	3.8%
	Myocyte vacuolization	5	1.3%
	Others (focal myocarditis 1, arteriolar wall thickening 1, focal myocardial infarct adjacent to bundle of His 1, cardiomegaly 2)	5	1.3%
	None or unclarified	299	63.6%
Lung	Pulmonary edema	247	55.9%
	Focal pulmonary hemorrhage	2	0.5%
	None or unclarified	141	36.1%

Interstitial fibrosis, myocyte disarray/hypertrophy, and mild atherosclerotic coronary artery disease were the most common symptoms of cardiac pathology, documented in 49 (12.7%), 18 (4.6%), and 15 cases (3.8%), respectively. Myocyte vacuolization was documented in 5 cases (1.3%). Other findings presented in five cases (1.3%) included focal myocarditis in one case, arteriolar wall thickening in one case, focal myocardial infarct adjacent to the bundle of His in one case, and cardiomegaly in two cases. A total of 299 (63.6%) cases had none or unclarified cardiac pathology.

Though it is non-specific to SUDEP and probably a result of any death, pulmonary congestion/edema comprised the most common finding of pulmonary pathology, documented in 247 cases (55.9%). Focal intra-alveolar hemorrhage was documented in two cases (0.5%). A total of 141 cases (36.1%) had none or unclarified pulmonary pathology.

## 4. Discussion

Sudden unexpected death in epilepsy was considered the leading cause of death in patients with epilepsy. By the common definition, the patients die suddenly with no anatomical or toxicological cause of death found, namely the negative postmortem examination. Diagnosis of SUDEP remains a difficult task for forensic pathologists (7, 31). Nevertheless, there is often a spectrum of pathological abnormalities among SUDEP individuals, including neuro- and cardiopulmonary pathological changes. To uncover such pathological changes, the present study pooled cases from our forensic center with literature-reported SUDEP cases and studied the neurological, cardiac, and pulmonary findings from a forensic autopsy perspective. The investigation yielded a total of 388 cases. All the SUDEP cases died at an early age (mostly 10–40 years), with the male-to-female ratio as 229:159. Neurological changes were the most common postmortem findings associated with SUDEP. Interstitial fibrosis, myocyte hypertrophy, and mild coronary artery atherosclerosis were the most common symptoms of primary cardiac pathology, and non-specific pulmonary edema/congestion was the major pulmonary finding.

The common neuropathological findings include mild degrees of cerebral edema or congestion, traumatic brain lesions, hippocampal sclerosis, vascular malformations, low-grade neoplasms, cerebellar atrophy, and cortical malformations (32, 33). The range of pathologies encompasses those commonly encountered in surgical epilepsy series, but no significant difference was shown in the frequency of neuropathological findings between the SUDEP cases and living patients with epilepsy (34). From a histological point of view, the most common finding was related to acute hypoxic neuronal changes, that is, eosinophilic neuronal changes, occurring in 55% of the SUDEP cases, most often in the hippocampus and also sometimes in the cortical and subcortical regions. Epilepsy-related acute hypoxic neuropathology in the brainstem may also contribute to the progression of epilepsy and eventually lead to brainstem dysfunction and cause SUDEP. These acute changes were more frequent when a seizure occurred 24 h before death, the body was in a prone position, or brain swelling was present (32–35). It is worth noting that, although many lesions may be identified, either grossly or microscopically, some epilepsy-related pathologies required specific immunohistochemistry to confirm the diagnosis (32, 33). In addition, published data suggested that neuropathology was very heterogeneous. The diverse neurological findings and heterogeneous data may reflect the non-standard method for conducting a brain examination across forensic institutes. It is thus important to highlight a standardized, widely occupied protocol for brain examination in suspected cases of SUDEP. The Royal College of Pathologists of United Kingdom (UK) issued guidelines in 2006 on autopsy practice in epilepsy death, suggesting that pathologists should have information on epilepsy, including seizure control, treatments, and the circumstantial evidence surrounding the death; a neuropathologist should be involved in the interpretation of brain pathology; and a case should be made for whole brain fixation and examination. A higher detection of intracerebral pathology was noted by examination of the whole fixed brain and examination of all essential regions microscopically compared with other methods (35). These guidelines are useful in forensic practice and may be recommended to be applied across different institutes to execute a uniform brain examination protocol.

Pulmonary pathology in SUDEP mainly consists of pulmonary edema/congestion and less often focal pulmonary hemorrhage. The two cases showing focal pulmonary hemorrhage may reflect asphyxia right before death due to SUDEP. Edema and pulmonary congestion are common autopsy findings of various cardiogenic or neurogenic deaths, including SUDEP (36). Confounding factors, such as postmortem interval, resuscitation with chest compression, and intravenous fluid administration as well as premorbid cardiopulmonary disease, may potentially contribute to the development of pulmonary edema (37). Therefore, pulmonary edema was a non-specific finding and may be of little association with SUDEP.

At the cardiac level, interstitial fibrosis, myocyte disarray/hypertrophy, myocyte vacuolization, and mild atherosclerotic coronary artery disease were mostly described in the literature. The increased frequency of cardiac findings in SUDEP may relate to the effects of seizures or psychotropic medications on the cardiac tissue (12, 38). Some studies argued that the presence and severity of cardiac pathology are not higher among SUDEP cases compared to age- and sex-matched controls who died from sudden cardiac death or trauma or to patients with epilepsy who died from causes other than SUDEP (39). From the forensic perspective, unremarkable coronary artery atherosclerosis, focal myocardial inflammatory infiltrates, isolated myocyte disarray, and idiopathic cardiac fibrosis were occasionally encountered by forensic pathologists, especially in case of sudden unexplained death. These changes, the so-called non-diagnostic autopsy findings, were insufficient to meet diagnostic criteria for known pathologies and insufficient to accord as causes for sudden death (40, 41). Although these subtle cardiac pathological findings could not prove the cause of death, a routine systemic histological examination of the myocardium, even in those who appear to be SUDEP cases, is warranted to monitor the significance of these unexpected findings. Furthermore, as regards the cardiac pathology among patients with epilepsy or SUDEP individuals, there is a need to obtain more information about the cardiorespiratory function in patients with epilepsy, which may contribute to better interpretation, and possibly, prevent SUDEP through interventions such as cardioprotective drugs and effective respiratory therapy.

Another important finding of the present study pertains to the varied postmortem levels of AEDs, ranging from the supra-therapeutic level to zero, mostly at sub-therapeutic concentration or being negative. Out of the 388 cases, 218 cases (56.2%) were at sub-therapeutic or negative levels of AEDs. Low levels of AEDs in postmortem blood have been proposed as a strong predictor of SUDEP, for low AED levels reflecting an inadequate dosage or non-compliance before death (42). However, some studies argued that detecting sub-therapeutic AED levels at autopsy has limited value in determining the cause of death, due to uncertainties in the correlation of postmortem whole blood levels with antemortem serum levels and the definition of a therapeutic level (43). In a recently published study comparing 13 SUDEP cases with 18 non-SUDEP forensic autopsy cases, the authors also found no significant difference between the two groups of cases with regard to the use of AEDs when considering postmortem toxicological results (44). Though the connection between sub-therapeutic or absent AEDs and the occurrence of SUDEP remains to be debated, the adjuvant use of antipsychotic drugs serving as an underlying mechanism of SUDEP received widespread attention. Psychiatric comorbidity is common in patients with epilepsy, and antipsychotic drugs may be prescribed more commonly among patients with epilepsy than in the general population (45). Antipsychotics could induce cardiac side effects, including heart rate changes, blood pressure alterations, and more severe and fatal issues, such as QTc prolongation, congestive heart failure, and even sudden unexpected death (46) *via* directly binding to cardiac CB1R (47) or disturbing spliceosome signaling (48). Therefore, it is necessary to evaluate the safety and efficiency of antipsychotic medications among patients with epilepsy, and their potential contribution toward SUDEP.

Our study has several limitations. First, due to a lack of information on individual race, we were unable to determine whether neurological, cardiac, and pulmonary pathologies have racial differences or not. Second, due to technical limitations, we failed to obtain sufficient information on patients' medical records, such as the clinical type of seizure. Collaborative studies involving both clinical physicians and forensic pathologists would be more helpful in illustrating the characteristics of SUDEP.

## 5. Conclusion

In all, we systemically analyzed the neurological, cardiac, and pulmonary pathology for SUDEP using cases from both our forensic center and literature resources. Neuropathology was the most common change for such cases. While all these changes do not explain the cause of death, our study might pave the way for understanding the pathogenesis of SUDEP and the interpretation of death. The present study also highlighted the standard examination of the vital organs in circumstances of such cases.

## Data availability statement

The original contributions presented in the study are included in the article/supplementary material, further inquiries can be directed to the corresponding author.

## Ethics statement

The studies involving human participants were reviewed and approved by Ethical Review Board at the School of Basic Medical Science, Gannan Medical University. The patients/participants provided their written informed consent to participate in this study. Written informed consent was obtained from the individual(s) for the publication of any potentially identifiable images or data included in this article.

## Author contributions

FY and YC were involved in the conception and design of the study and wrote the original draft. FZ, YY, and LZ were involved in the forensic data collection, literature search, and data analysis. All authors have read and agreed to the published version of the manuscript.
